# Supplementary material for: Resolving the full spectrum of human genome variation using Linked-Reads
Source: Genome Res. 2019 Apr;29(4):635–45. doi: 10.1101/gr.234443.118 (PMC6442396; doi:10.1101/gr.234443.118)
Supplement: Supplemental Material [file supp_gr.234443.118_Supplemental_longranger-2.2.zip › longranger-2.2.tar.bz2!/lr2.2-public/lib/stan/cmdstan/stan/doc/api/html/classstan_1_1mcmc_1_1base__hamiltonian.html]

Stan: stan::mcmc::base\_hamiltonian< M, P, BaseRNG > Class Template Reference


|  |  |
| --- | --- |
|  | Stan  2.5.0  probability, sampling & optimization |


- Main Page
- Related Pages
- Namespaces
- Classes
- Files

- Class List
- Class Index
- Class Hierarchy
- Class Members

All Classes Namespaces Files Functions Variables Typedefs Enumerations Enumerator Friends Macros Pages

- stan
- mcmc
- base\_hamiltonian

Public Member Functions |
Protected Member Functions |
Protected Attributes |
List of all members

stan::mcmc::base\_hamiltonian< M, P, BaseRNG > Class Template Referenceabstract

`#include <base_hamiltonian.hpp>`

|  |  |
| --- | --- |
| Public Member Functions | |
|  | base\_hamiltonian (M &m, std::ostream \*e) |
|  | |
|  | ~base\_hamiltonian () |
|  | |
| virtual double | T (P &z)=0 |
|  | |
| double | V (P &z) |
|  | |
| virtual double | tau (P &z)=0 |
|  | |
| virtual double | phi (P &z)=0 |
|  | |
| double | H (P &z) |
|  | |
| virtual const Eigen::VectorXd | dtau\_dq (P &z)=0 |
|  | |
| virtual const Eigen::VectorXd | dtau\_dp (P &z)=0 |
|  | |
| virtual const Eigen::VectorXd | dphi\_dq (P &z)=0 |
|  | |
| virtual void | sample\_p (P &z, BaseRNG &rng)=0 |
|  | |
| virtual void | init (P &z) |
|  | |
| virtual void | update (P &z) |
|  | |

|  |  |
| --- | --- |
| Protected Member Functions | |
| void | write\_error\_msg\_ (std::ostream \*error\_msgs, const std::exception &e) |
|  | |

|  |  |
| --- | --- |
| Protected Attributes | |
| M & | model\_ |
|  | |
| std::ostream \* | err\_stream\_ |
|  | |

## Detailed Description

### template<typename M, typename P, typename BaseRNG> class stan::mcmc::base\_hamiltonian< M, P, BaseRNG >

Definition at line 17 of file base\_hamiltonian.hpp.

## Constructor & Destructor Documentation

template<typename M, typename P, typename BaseRNG>

|  |  |  |  |
| --- | --- | --- | --- |
| stan::mcmc::base\_hamiltonian< M, P, BaseRNG >::base\_hamiltonian | ( | M & | *m*, |
|  |  | std::ostream \* | *e* |
|  | ) |  |  |

inline

Definition at line 21 of file base\_hamiltonian.hpp.

template<typename M, typename P, typename BaseRNG>

|  |  |  |  |  |
| --- | --- | --- | --- | --- |
| stan::mcmc::base\_hamiltonian< M, P, BaseRNG >::~base\_hamiltonian | ( |  | ) |  |

inline

Definition at line 22 of file base\_hamiltonian.hpp.

## Member Function Documentation

template<typename M, typename P, typename BaseRNG>

|  |  |  |  |  |  |
| --- | --- | --- | --- | --- | --- |
| virtual const Eigen::VectorXd stan::mcmc::base\_hamiltonian< M, P, BaseRNG >::dphi\_dq | ( | P & | *z* | ) |  |

pure virtual

Implemented in stan::mcmc::dense\_e\_metric< M, BaseRNG >, stan::mcmc::diag\_e\_metric< M, BaseRNG >, and stan::mcmc::unit\_e\_metric< M, BaseRNG >.

template<typename M, typename P, typename BaseRNG>

|  |  |  |  |  |  |
| --- | --- | --- | --- | --- | --- |
| virtual const Eigen::VectorXd stan::mcmc::base\_hamiltonian< M, P, BaseRNG >::dtau\_dp | ( | P & | *z* | ) |  |

pure virtual

Implemented in stan::mcmc::dense\_e\_metric< M, BaseRNG >, stan::mcmc::diag\_e\_metric< M, BaseRNG >, and stan::mcmc::unit\_e\_metric< M, BaseRNG >.

template<typename M, typename P, typename BaseRNG>

|  |  |  |  |  |  |
| --- | --- | --- | --- | --- | --- |
| virtual const Eigen::VectorXd stan::mcmc::base\_hamiltonian< M, P, BaseRNG >::dtau\_dq | ( | P & | *z* | ) |  |

pure virtual

Implemented in stan::mcmc::dense\_e\_metric< M, BaseRNG >, stan::mcmc::diag\_e\_metric< M, BaseRNG >, and stan::mcmc::unit\_e\_metric< M, BaseRNG >.

template<typename M, typename P, typename BaseRNG>

|  |  |  |  |  |  |
| --- | --- | --- | --- | --- | --- |
| double stan::mcmc::base\_hamiltonian< M, P, BaseRNG >::H | ( | P & | *z* | ) |  |

inline

Definition at line 30 of file base\_hamiltonian.hpp.

template<typename M, typename P, typename BaseRNG>

|  |  |  |  |  |  |
| --- | --- | --- | --- | --- | --- |
| virtual void stan::mcmc::base\_hamiltonian< M, P, BaseRNG >::init | ( | P & | *z* | ) |  |

inlinevirtual

Definition at line 41 of file base\_hamiltonian.hpp.

template<typename M, typename P, typename BaseRNG>

|  |  |  |  |  |  |
| --- | --- | --- | --- | --- | --- |
| virtual double stan::mcmc::base\_hamiltonian< M, P, BaseRNG >::phi | ( | P & | *z* | ) |  |

pure virtual

Implemented in stan::mcmc::dense\_e\_metric< M, BaseRNG >, stan::mcmc::diag\_e\_metric< M, BaseRNG >, and stan::mcmc::unit\_e\_metric< M, BaseRNG >.

template<typename M, typename P, typename BaseRNG>

|  |  |  |  |
| --- | --- | --- | --- |
| virtual void stan::mcmc::base\_hamiltonian< M, P, BaseRNG >::sample\_p | ( | P & | *z*, |
|  |  | BaseRNG & | *rng* |
|  | ) |  |  |

pure virtual

Implemented in stan::mcmc::dense\_e\_metric< M, BaseRNG >, stan::mcmc::diag\_e\_metric< M, BaseRNG >, and stan::mcmc::unit\_e\_metric< M, BaseRNG >.

template<typename M, typename P, typename BaseRNG>

|  |  |  |  |  |  |
| --- | --- | --- | --- | --- | --- |
| virtual double stan::mcmc::base\_hamiltonian< M, P, BaseRNG >::T | ( | P & | *z* | ) |  |

pure virtual

Implemented in stan::mcmc::dense\_e\_metric< M, BaseRNG >, stan::mcmc::diag\_e\_metric< M, BaseRNG >, and stan::mcmc::unit\_e\_metric< M, BaseRNG >.

template<typename M, typename P, typename BaseRNG>

|  |  |  |  |  |  |
| --- | --- | --- | --- | --- | --- |
| virtual double stan::mcmc::base\_hamiltonian< M, P, BaseRNG >::tau | ( | P & | *z* | ) |  |

pure virtual

Implemented in stan::mcmc::dense\_e\_metric< M, BaseRNG >, stan::mcmc::diag\_e\_metric< M, BaseRNG >, and stan::mcmc::unit\_e\_metric< M, BaseRNG >.

template<typename M, typename P, typename BaseRNG>

|  |  |  |  |  |  |
| --- | --- | --- | --- | --- | --- |
| virtual void stan::mcmc::base\_hamiltonian< M, P, BaseRNG >::update | ( | P & | *z* | ) |  |

inlinevirtual

Definition at line 43 of file base\_hamiltonian.hpp.

template<typename M, typename P, typename BaseRNG>

|  |  |  |  |  |  |
| --- | --- | --- | --- | --- | --- |
| double stan::mcmc::base\_hamiltonian< M, P, BaseRNG >::V | ( | P & | *z* | ) |  |

inline

Definition at line 25 of file base\_hamiltonian.hpp.

template<typename M, typename P, typename BaseRNG>

|  |  |  |  |
| --- | --- | --- | --- |
| void stan::mcmc::base\_hamiltonian< M, P, BaseRNG >::write\_error\_msg\_ | ( | std::ostream \* | *error\_msgs*, |
|  |  | const std::exception & | *e* |
|  | ) |  |  |

inlineprotected

Definition at line 63 of file base\_hamiltonian.hpp.

## Member Data Documentation

template<typename M, typename P, typename BaseRNG>

std::ostream\* stan::mcmc::base\_hamiltonian< M, P, BaseRNG >::err\_stream\_

protected

Definition at line 61 of file base\_hamiltonian.hpp.

template<typename M, typename P, typename BaseRNG>

M& stan::mcmc::base\_hamiltonian< M, P, BaseRNG >::model\_

protected

Definition at line 59 of file base\_hamiltonian.hpp.

---

The documentation for this class was generated from the following file:

- src/stan/mcmc/hmc/hamiltonians/base\_hamiltonian.hpp


---

[ Stan Home Page ]

*© 2011–2014,
Stan Development Team.*
